# *Operando* Raman spectroscopy for investigating lithium deposition/dissolution and diffusion at the microelectrode surface†

**DOI:** 10.1039/d5ra03080c

**Published:** 2025-07-10

**Authors:** Hayate Mukofukasawa, Koji Hiraoka, Shiro Seki

**Affiliations:** a Graduate School of Applied Chemistry and Chemical Engineering, Kogakuin University 2665-1 Nakano-machi Hachioji-shi Tokyo 192-0015 Japan shiro-seki@cc.kogakuin.ac.jp

## Abstract

We developed an *operando* Raman spectroscopy method to visualize lithium deposition and diffusion at the microelectrode interface. Lithium deposition released free EC and FSA, while dissolution promoted rapid Li^+^ coordination. The estimated diffusion layer thickness matched theoretical predictions, highlighting micro-Raman spectroscopy's potential for studying lithium-ion battery interfaces.

## Abstract 2

*Operando* Raman spectroscopy was developed to visualize interfacial reactions at a microelectrode, revealing environmental changes during Li deposition/dissolution and enabling analysis of dynamic battery interface processes.

## Graphical abstract

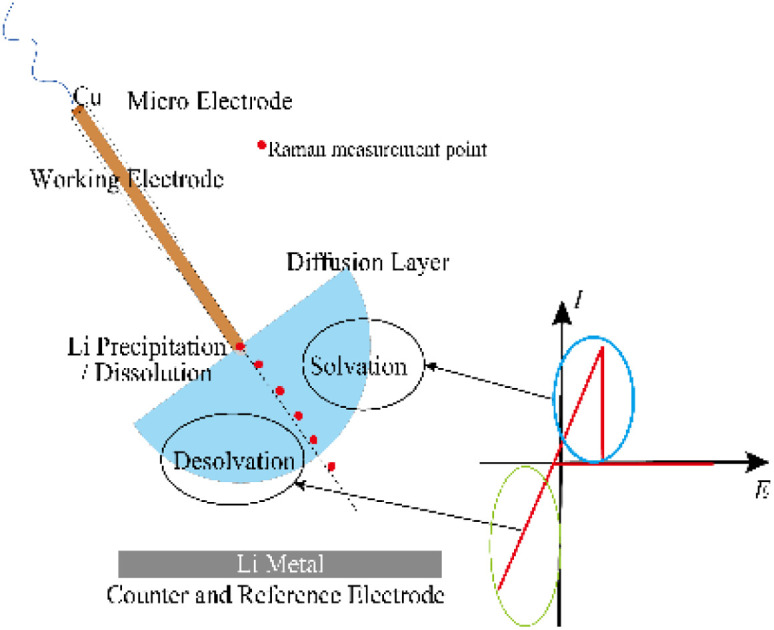

The efficient utilization of renewable energy has become an urgent global priority, driving extensive research into energy storage technologies that leverage cost-effective and highly safe battery systems.^1–3^ Among these, lithium-ion batteries (LIBs) have emerged as a dominant energy storage solution due to their high energy density, particularly in applications related to the electrification of transportation.^4,5^ A conventional LIB comprises positive and negative electrode, and electrolyte that facilitates the migration of lithium ions (Li^+^) between electrodes, enabling charge transfer and energy storage.^6,7^ The electrolyte typically consists of an organic carbonate solvent containing approximately 1.0 mol kg^−1^ of lithium salt.^8,9^ At the electrode–electrolyte interface, charge transfer occurs *via* the formation of an electrical double layer, while Li^+^ ions undergo diffusion within the electrolyte, forming a concentration gradient near the electrode surface. The diffusion behaviour of Li^+^ ions is commonly analysed using Fick's laws of diffusion, and recent advances in computational chemistry have facilitated the quantitative evaluation of Li^+^ transport dynamics, offering deeper insights into ion migration mechanisms in LIBs.^10–13^ Electrode reactions in LIBs are governed by charge transfer processes occurring at the electrode–electrolyte interface.^10^ These reactions are primarily influenced by three factors: (1) the kinetics of electron transfer, (2) the diffusion of reactive species at the electrode surface, and (3) mass transport within the electrolyte.^14^ Under typical conditions, the reaction rate is largely dictated by electron transfer kinetics, often exhibiting an exponential dependence on the electrode potential.^15^ However, under diffusion-limited conditions, as in typical LIBs, the reaction rate is governed by mass transport constraints.^16^

The diffusion behaviour of ionic species in electrochemical systems is generally described by Fick's first and second laws.^10^ Electrochemical reactions induce localized concentration gradients near the electrode surface, leading to the formation of a diffusion layer.^10^ In planar electrode configurations, mass transport is predominantly controlled by semi-infinite one-dimensional diffusion, where the rate at which reactants reach the electrode surface determines the overall reaction kinetics. As electrode dimensions decrease, the geometric characteristics of diffusion undergo significant changes.^16^ In microelectrodes with submicron diameters, diffusion occurs in a spherical or radial manner. Under these conditions, mass transport becomes the rate-limiting step as it is slower than the electrode reaction kinetics, amplifying the influence of diffusion-limited kinetics. In the short-time regime of potential step experiments, Cottrell's equation governs the transient current response under diffusion-controlled conditions. In contrast, at longer timescales, the system attains a steady-state condition, where the current stabilizes and remains independent of time.^17^

Enhancing LIB performance necessitates a comprehensive understanding of degradation mechanisms and a detailed analysis of the temporal evolution of electrode reactions. Analytical methodologies for investigating these processes can be broadly categorized into *in situ* and *ex situ* techniques, including *operando* methods, enable real-time monitoring of batteries during charge–discharge cycles.^7,8^*In situ* methods enable real-time monitoring of batteries during charge–discharge cycles, with spectroscopic techniques such as Raman spectroscopy widely utilized to examine Li^+^ insertion into graphite and ion transport behaviour.^18^ Additional analytical approaches involve the selective substitution of active material in sheet electrodes with electrochemically inert alumina to facilitate electrochemically evaluating the Li^+^ diffusion process. Alternatively, single-particle electrochemical characterization using a microprobe provides valuable insights into the intrinsic properties of active materials.^19,20^ These methodologies enable the determination of activation energy for electrode reactions, contributing to a fundamental understanding of LIB kinetics. Furthermore, computational chemistry techniques have become indispensable for simulating electrode surface reactions, offering valuable insights into the underlying electrochemical mechanisms.^21–23^ Despite the widespread application of *in situ* analytical techniques, direct observations at the microscopic scale of electrode reactions remain scarce. In this study, we have developed an advanced Raman spectroscopic methodology that enables direct visualization of electrode–electrolyte interactions using a microprobe. This novel approach facilitates high-spatial-resolution investigations of interfacial electrochemical processes, offering new insights into the fundamental reaction mechanisms governing LIB performance.

To prepare the microelectrode for measurements, a glass capillary (G-100L, inside diameter: 0.75 mm, Narishige) was heated and pulled using a puller (PC-10, Narishige), resulting in an acute apex. Thin Pt wire (*ϕ* 30 μm, Nilaco) and thick Cu wire (*ϕ* 100 μm, Nilaco) were welded together using a spot-welding machine (LTH-MWS, Kondo Tech) to obtain self-standing metal wires. The hybridized wire was inserted into the melted apex of the glass capillary and fused using a microforge (MF-900, Narishige). To ensure accurate electrical contact, the microelectrode tip was polished at a 45° angle using a rotary polishing machine (EG-402, Narishige). For *operando* Raman spectroscopic measurements of the electrolyte, the equipment shown in Fig. 1(a) was installed in a dry-box (DPA-02A, ORION Machinery, [H_2_O] < 10 ppm). A micromanipulator (QP-3EH, Micro Support) was used to specify the measurement position along the *X*- and *Y*-axes, and *Z*-axis was fixed. A microscope-attached Raman spectrometer (RMP-510, JASCO & OLYMPUS) and an electrochemical measurement device (SP-200, Bio-Logic) were also set up.

**Fig. 1:**
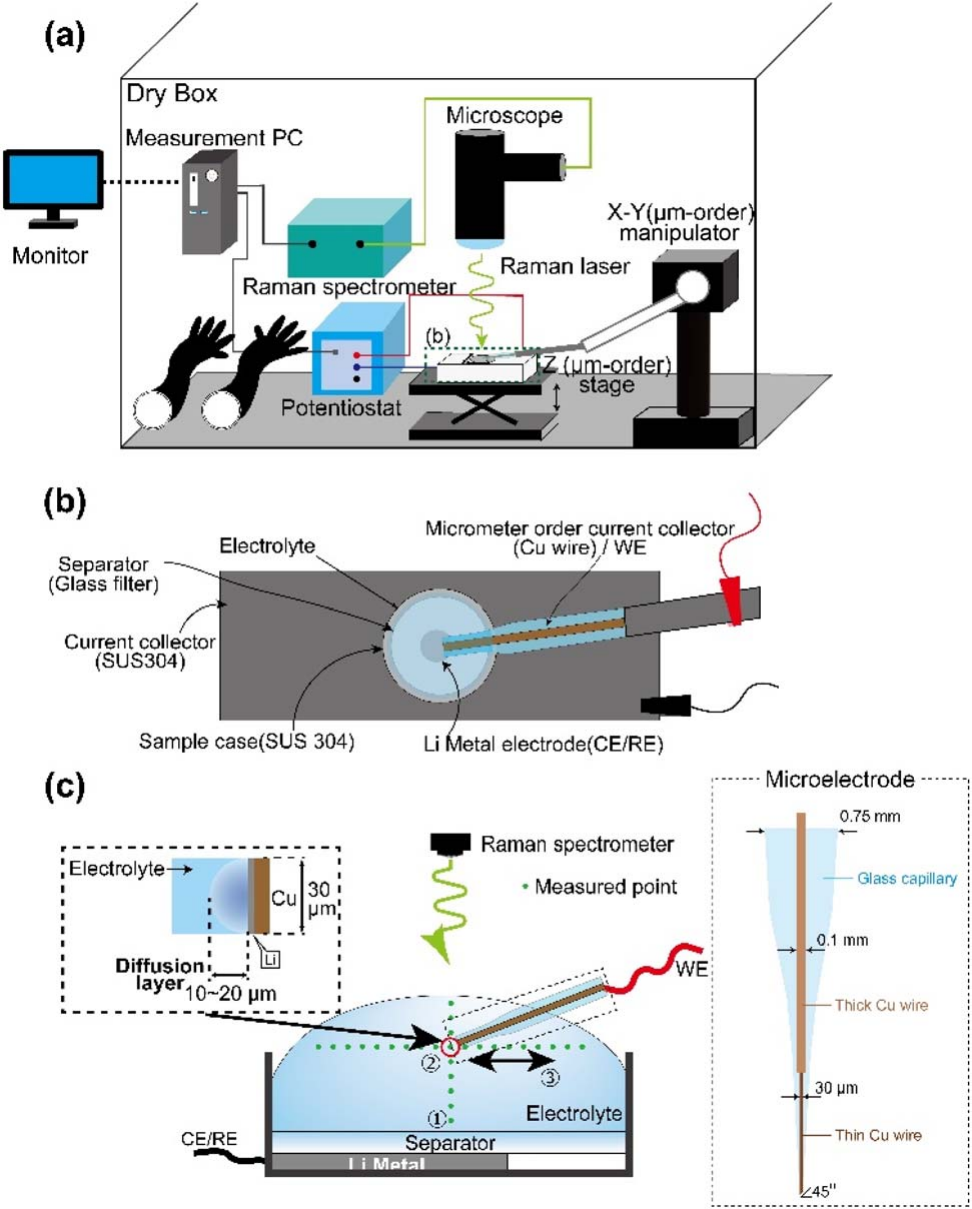
Schematic image of *operando* Raman measurement setup (a), top view of *operando* Raman measurement cell (b), and schematic image of *operando* Raman measurements with microelectrode illustration (c).

As shown in Fig. 1(b), a Li foil (counter/reference electrode, *ϕ* 3 mm) was affixed to a SUS cup, and a separator impregnated with the electrolyte (1.0 mol per kg EC (ethylene carbonate)/LiFSA solution) was placed inside. A prepared micro-scale Cu current collector, and its tip immersed in the electrolyte. This micro Cu electrode served as the working electrode, and measurements were performed at room-temperature using the electrochemical measurement device. Measurement positions for the *operando* Raman spectroscopy are also shown in Fig. 1(c). Measurements were conducted under three patterns as 1 to 3 with identical conditions: grating 2400 cm^−1^, slit *ϕ* 100 μm, measurement wavenumber range 400–1500 cm^−1^, objective lens ×50, leaser diameter *ϕ* 1 μm, and resolution 4.77 cm^−1^.

Pattern 1: to determine the vertical (*Z*-axis) focus and correct the measurement distance from the microelectrode surface, Raman spectra were recorded while adjusting the *z*-position. The reference height was defined as the point where the probe tip was in focus during microscope observation. The probe was moved within a range of −100 μm to 100 μm in the vertical direction, and Raman spectra was acquired every 10 μm to obtain the clearest spectra. Raman measurements were performed after the current stabilized, approximately 5 min after each movement.

Pattern 2: to observe waveforms differences in Raman spectra at various potentials, potential sweep measurements were performed from open-circuit voltage (OCV) to −100 mV with scan rate of 0.10 mV s^−1^, and Raman spectra were acquired at each potential.

Pattern 3: to investigate waveform differences in measurement positions along the *X*-axis of the electrode under constant potential conditions (−25 mV), Raman measurements were performed at 5 μm intervals. Raman spectra were acquired along the *X*-axis, and curve fitting was applied to the peak near 900 cm^−1^. The fitted peaks were assigned to free EC and Li-bound EC, and the area ratio of the two peaks was calculated and analysed for changes of solvation state into electrolyte solution.

Fig. 2 shows the changes of Raman spectra during the potential sweeping of the electrolyte (pattern 2). Noted that all range of the obtained Raman spectra are shown in Fig. S1.† The microscopic image of the probe tip is also indicated in Fig. 2, and the distance from the electrode surface to the measurement point was determined to be 3.7 μm based on the image analysis. In the Raman spectra, the peak observed in the range of 885–900 cm^−1^ was assigned to ‘free’ ethylene carbonate (EC), which is not coordinated with Li^+^, whereas the peaks appearing in the range of 900–915 cm^−1^ correspond to ‘bound’ EC, in which Li^+^ is coordinated with EC. As the potential decreases, intensity of the free EC peak increases, while that of the Li-bound EC decreases. To differentiate between these spectral components, curve fitting was performed for each applied potential, enabling the deconvolution of the free EC and Li-bound EC peaks. The example of the fitting profiles is illustrated in Fig.S2.† From the fitting results, the peak areas corresponding to free EC and Li-bound EC were quantified, and the relative peak area ratios (*x*) were calculated. The relationship between the applied potential and the area ratio is presented in Fig. S3† and 3(a). Fig. S4† displays the full range of measured potentials, while Fig. 3(a) focuses on the potential range from −150 mV to 300 mV *vs.* Li/Li^+^. Similarly, curve fitting was also applied to the spectral range of 700 to 750 cm^−1^, corresponding to the SNS vibrational mode of the bis(fluorosulfonyl)amide (FSA) anion, to analyse changes in the area ratio. The relationship between the applied potential and the area ratio of the FSA peaks is illustrated in Fig. S3† and 3(b). Fig. 3(b) also presents the variation of free and bond FSA. In Fig. 3(a), (b) and S4,† the vertical axis (denoted as *x*) represents the peak area ratio of free and Li-bound species.

**Fig. 2:**
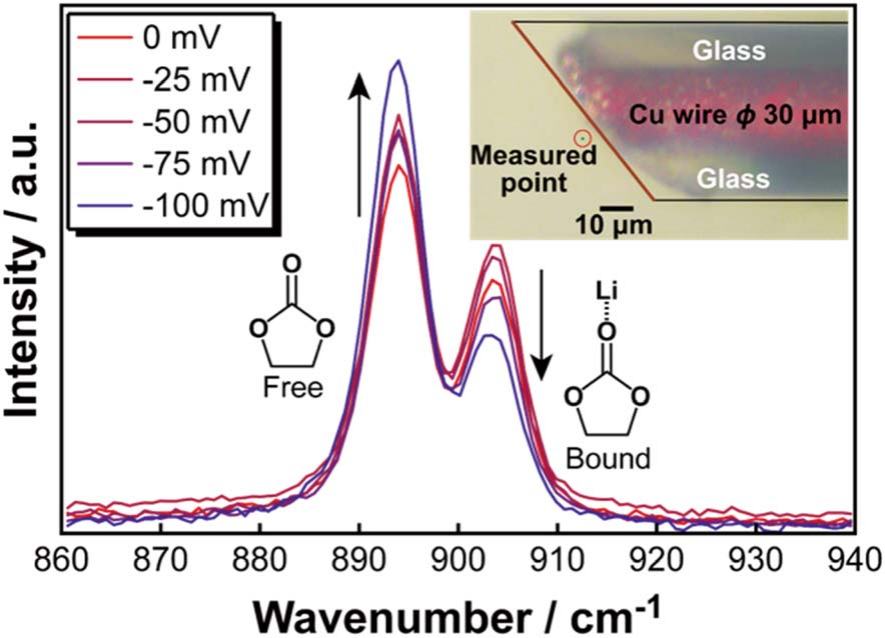
Raman spectra changes during the potential sweeping of the 1.0 mol per kg EC–LiFSA electrolyte.

**Fig. 3:**
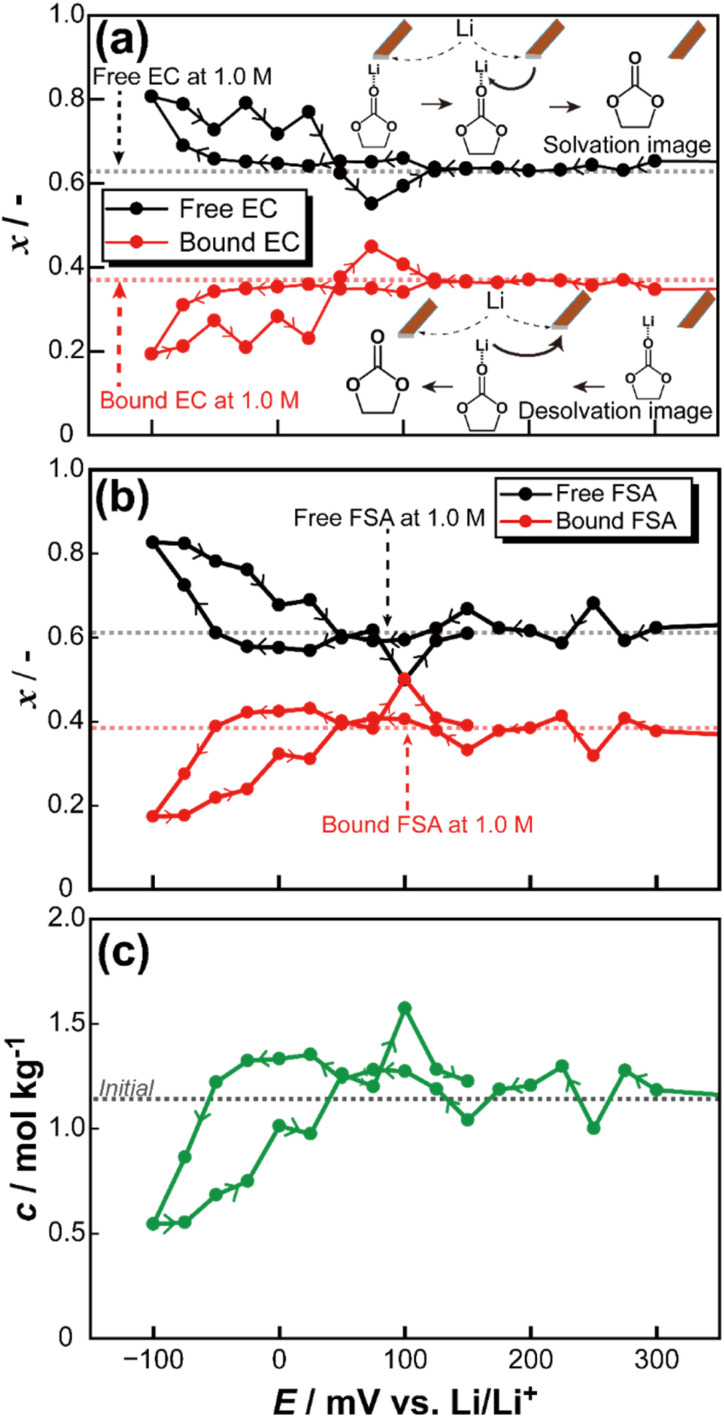
Fraction change of free and Li-bound EC and schematic image of Li solvation–desolvation (a), fraction change of free and Li-bound FSA (b), and concentration changes at electrode surface during potential sweeping (c).

Within the potential range (mainly, less than 0 V *vs.* Li/Li^+^) where lithium deposition occurs, an increase in the proportion of free EC and free FSA was observed. This phenomenon is attributed to the release of lithium ions from Li-bound EC and FSA during lithium deposition process. In contrast, upon potential reversal and the initiation of lithium dissolution, changes in the Raman spectra were evident, accompanied by a transient increase in the peak area ratio of Li-bound EC and FSA. This behaviour suggests that the dissolved lithium ions rapidly coordinate with free EC and free FSA. To further assess the local concentration variations associated with these spectral changes, experimental Raman intensity values were converted into concentration values using the calibration data shown in Fig. S5.† The relationship between the applied potential and local lithium concentration is depicted in Fig. 3(c). During the potential sweep, the lithium concentration near the deposition peak was approximately 0.5 mol kg^−1^, whereas near the dissolution peak, it increased to approximately 1.6 mol kg^−1^.

Fig. 4(a) illustrates the relationship between the peak area ratio of Raman spectra (Fig. S6†) and the distance from the electrode surface (pattern 3). Microscopic image analysis confirmed that the initial measurement point was positioned 1.7 μm from the electrode surface. At the electrode surface, lithium deposition induced a transient increase in the fraction of free EC and free FSA. Subsequently, for FSA, the ratio of free to Li-bound species inverted, and this trend persisted up to 35 μm from the electrode surface. In contrast, the peak area ratio of EC returned to its initial state at approximately 11 μm from the electrode surface. To theoretically analyse these findings, the diffusion layer thickness (*δ*) was estimated based on Fick's first law and the steady-state diffusion-limited current equation. According to Fick's first law, the diffusion flux *J* is given by:

1 *J* = −*D*(d*c*/d*x*)

where *D* is the diffusion coefficient, and (d*c*/d*x*) represents the concentration gradient. For a microdisc electrode, the steady current *I* is expressed as:

2 *I* = 4*nFDcr*

where *n* is the number of electrons involved in the reaction, *F* is Faraday's constant, *c* is the bulk concentration of the diffusing species, and *r* is the electrode radius. By converting the flux equation into current density *i*, we obtain:

3 *i* = *nFJ* = −*nFD*(d*c*/d*x*)

**Fig. 4:**
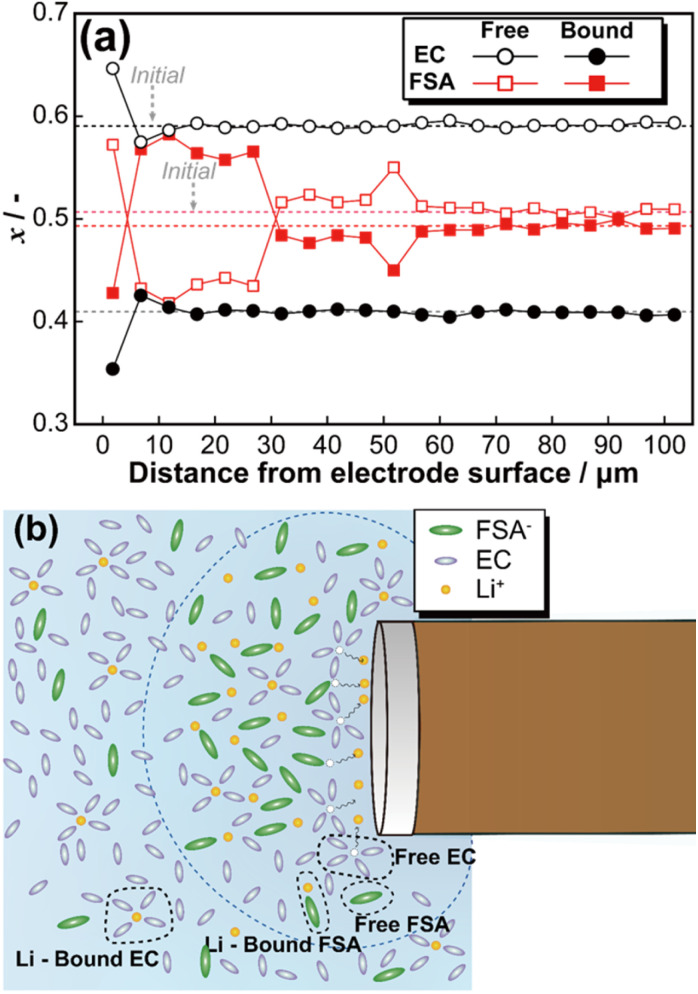
Fraction change of free and Li-bound EC and FSA during constant voltage at −25 mV (a), and schematic image of diffusion layer (b).

Assuming that at the electrode surface (*x* = 0), the concentration (*c*) is reduced to zero due to the electrochemical reaction, and at the diffusion layer boundary (*x* = *δ*), the concentration is *c*, the current density can be rewritten as:

4 *i* = *nFD*(−*c*/*δ*) = *nFDc*/*δ*

Similarly, dividing the steady-state current equation by the surface area (π*r*^2^) of the microdisk electrode, we get:

5 *i* = 4*nFDcr*/π*r*^2^ = 4*nFDc*/π*r*

Equating the two expressions for *i*, we derive the diffusion layer thickness:

6 *δ* = π*r*/4

Since the electrode used in this study has a diameter of 30 μm, substitution into eqn (6) yields a corresponding value of approximately 11 μm. This result exhibits excellent agreement with the variation in EC observed in Fig. 4(a). Thus, micro-Raman spectroscopy of the EC electrolyte concentration changes provides a means to estimate the thickness of the diffusion layer. Based on these findings, Fig. 4(b) presents a schematic representation of the diffusion layer at the microelectrode surface. Lithium dissolution at the electrode surface led to the accumulation of Li-bound EC in the vicinity of the electrode, resulting in a decrease in the fraction of free EC. Additionally, at the microelectrode surface, the fraction of Li-bound FSA was higher than that of free FSA. Also, the linearity between the free/bound EC and FSA ratio and electrolyte concentration appears to deviate above 0.8 mol kg^−1^, which may lead to overestimation of local concentrations in *operando* measurements. Further refinement of the model will be pursued through detailed investigations of the electrolyte system.

The *operando* Raman spectroscopy system developed in this study successfully enabled the direct observation of diffusion phenomena at the microelectrode surface. Unlike conventional computational techniques, this methodology allows real-time spectroscopic visualization of lithium deposition and dissolution processes. These results demonstrate the potential of micro-Raman spectroscopy as a powerful tool for investigating interfacial electrochemical phenomena in LIB systems.

## Conflicts of interest

The authors declare there are no conflicts of interest.

## Supplementary Material

RA-015-D5RA03080C-s001

## Data Availability

The data supporting this article have been included as part of the ESI.
