# Supplementary material for: Operando Raman spectroscopy for investigating lithium deposition/dissolution and diffusion at the microelectrode surface
Source: RSC Adv. 2025 Jul 10;15(30):24117–21. doi: 10.1039/d5ra03080c (PMC12243104; doi:10.1039/d5ra03080c)
Supplement: RA-015-D5RA03080C-s001 [file RA-015-D5RA03080C-s001.pdf]

Supplementary Information (SI) for RSC Advances.
This journal is © The Royal Society of Chemistry 2025

Electronic Supplementary Information

Operando Raman spectroscopy for investigating lithium deposition / dissolution and diffusion at the microelectrode surface

*Hayate Mukofukasawa, Koji Hiraoka and Shiro Seki**

Graduate School of Applied Chemistry and Chemical Engineering, School of Advanced Engineering, Kogakuin University, 2665-1 Nakano-machi, Hachioji, Tokyo 192-0015, Japan

*To whom correspondence should be addressed, E-mail: shiro-seki@cc.kogakuin.ac.jp (Dr. S. Seki)

Fax: +81-42-628-4568; Tel.: +81-42-628-4568

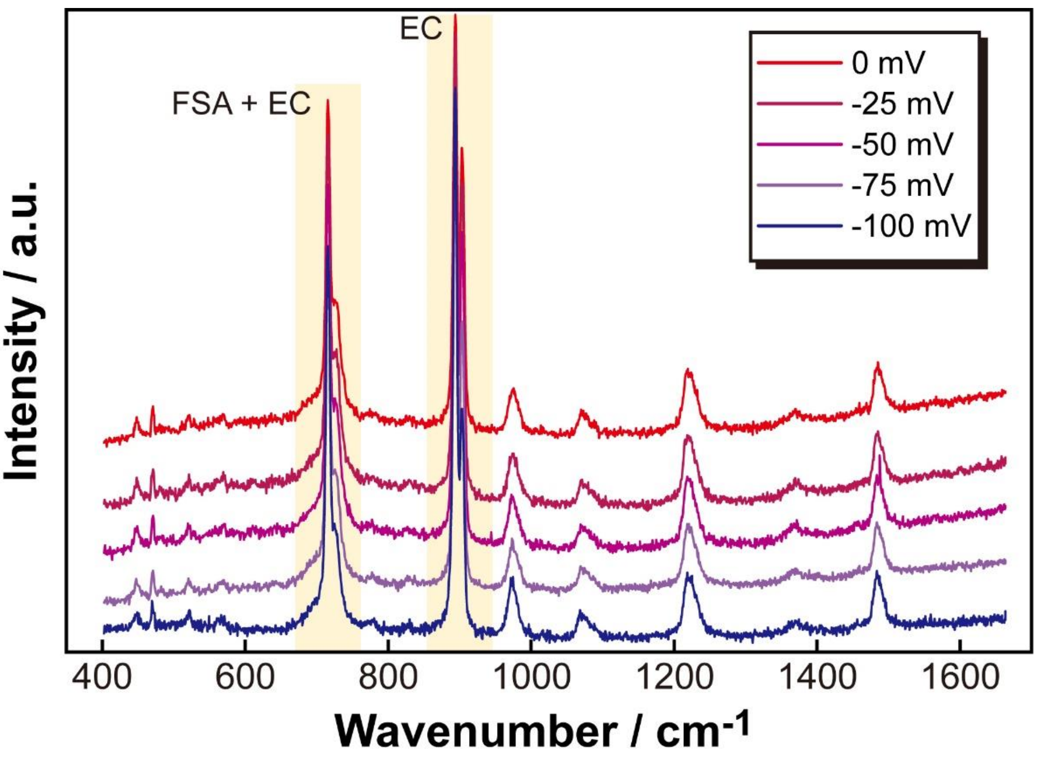

Fig. S1. All range of obtained Raman spectra during the potential sweep.

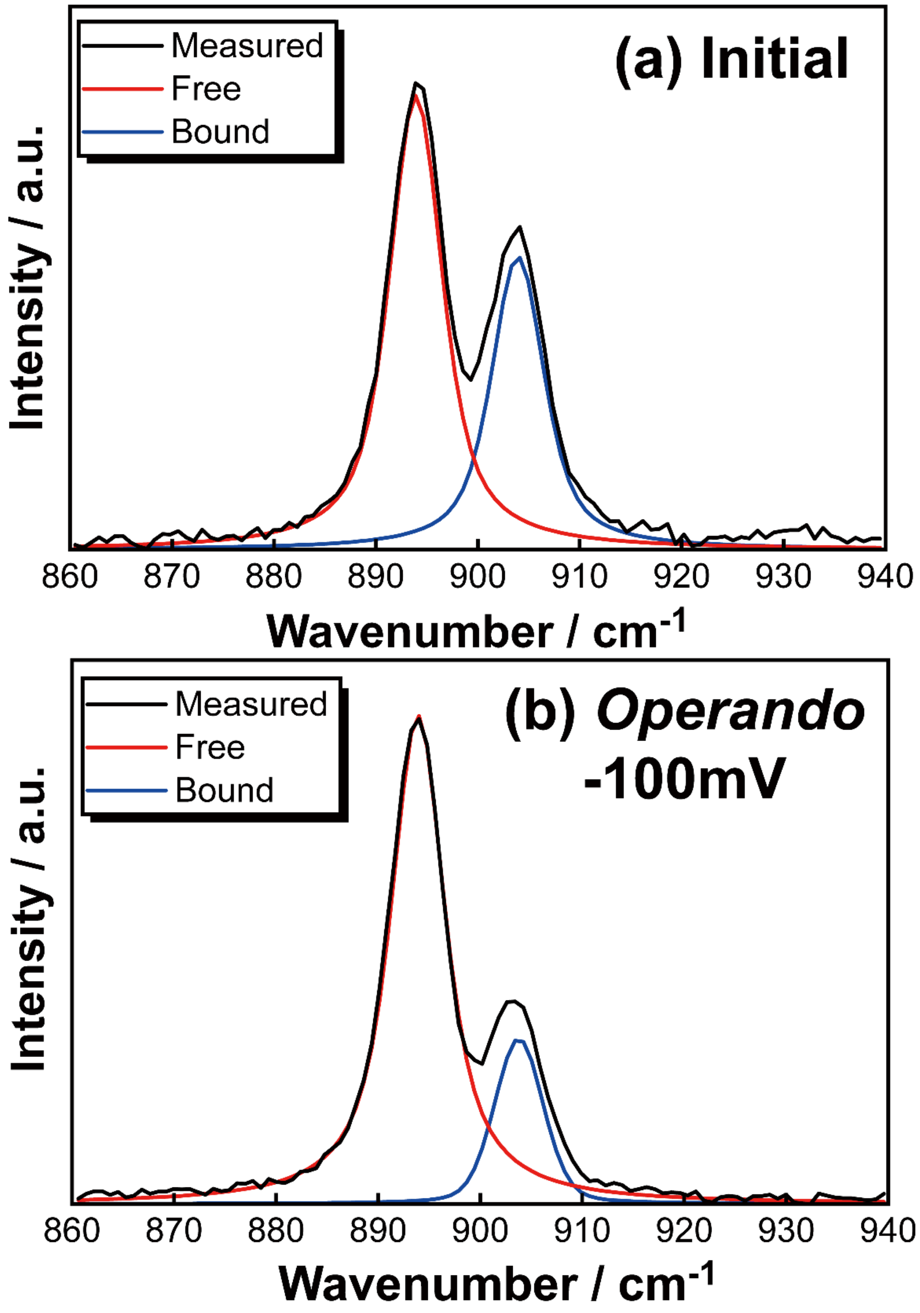

Fig. S2. Raman spectra of the EC-LiFSA electrolyte with a concentration of 1.0 mol kg^{-1} (a) and spectra acquired under operando conditions at -100 mV vs. Li/Li^{+}.

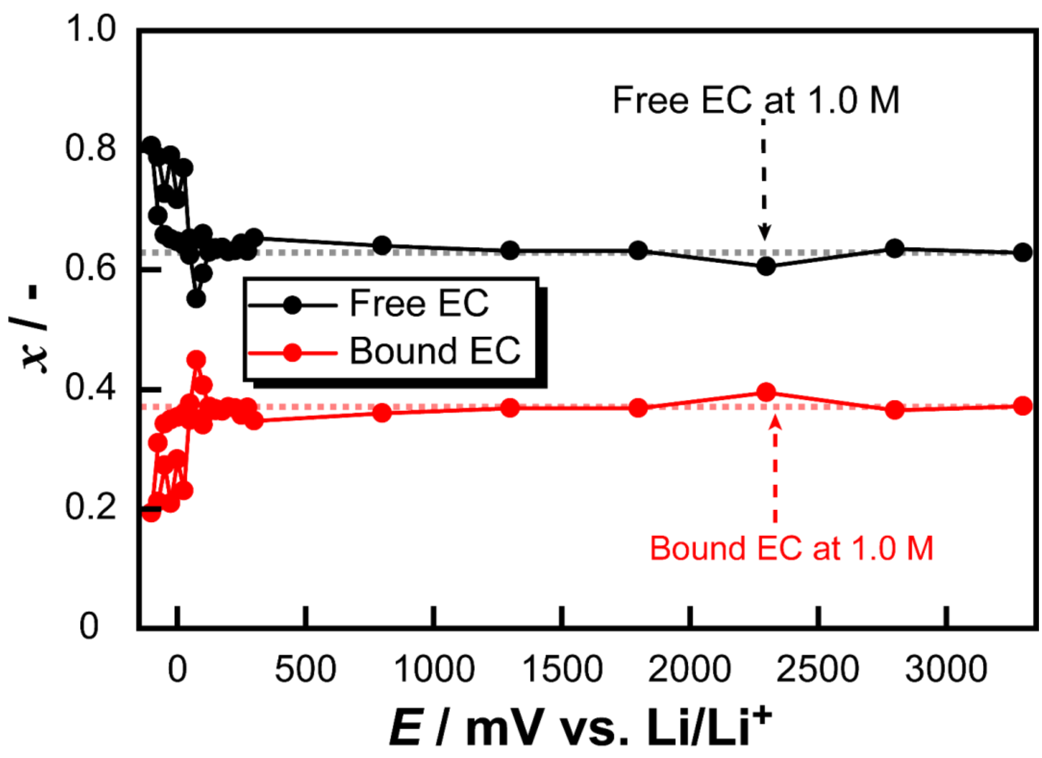

Fig. S3 Evolution of the peal area ratio of free EC and bound EC during the electrochemical potential sweep.

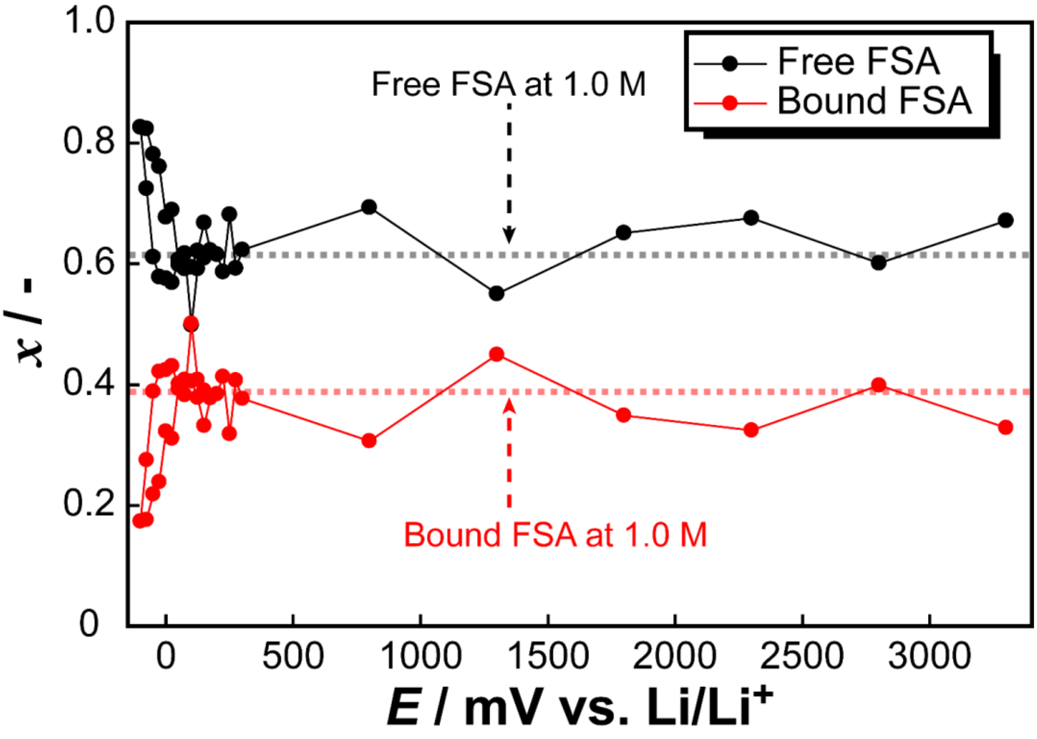

Fig. S4. Evolution of the peak area ratio of free FSA and bound FSA during the electrochemical potential sweep.

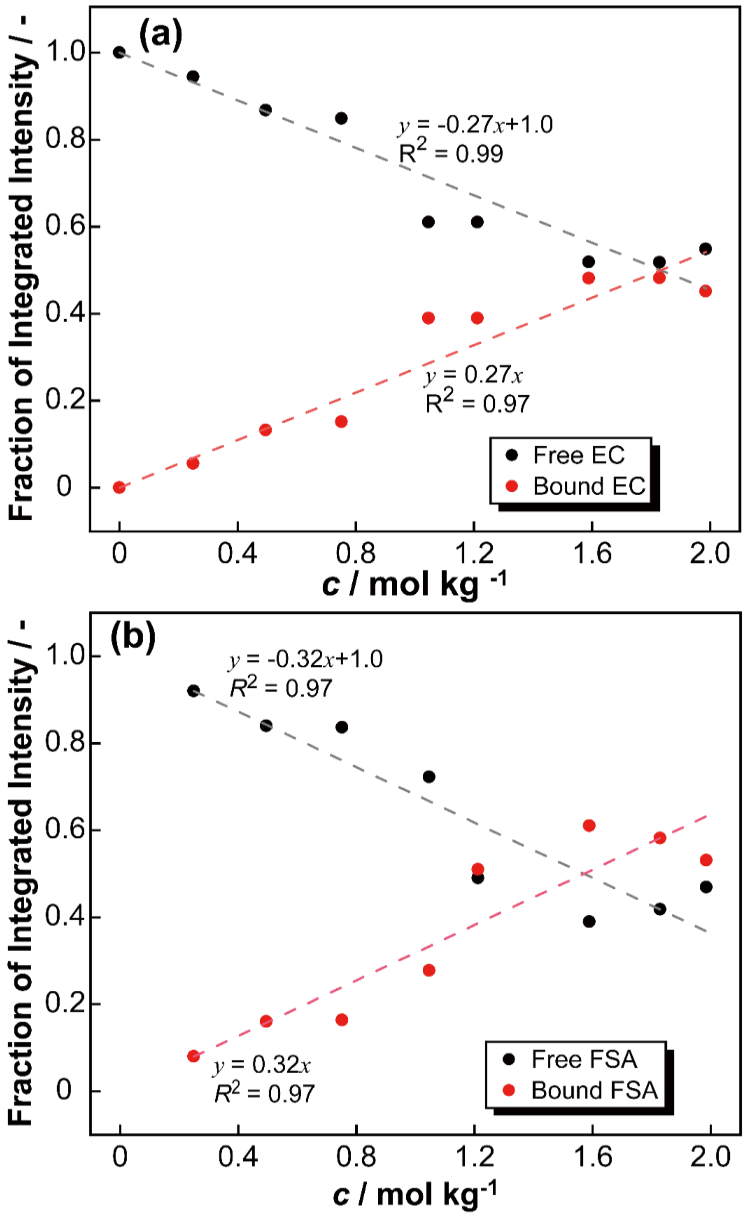

Fig. S5. Variations in the peak area ratio of free EC and bound EC (a), and free FSA and bound FSA (b) at different electrolyte concentrations.

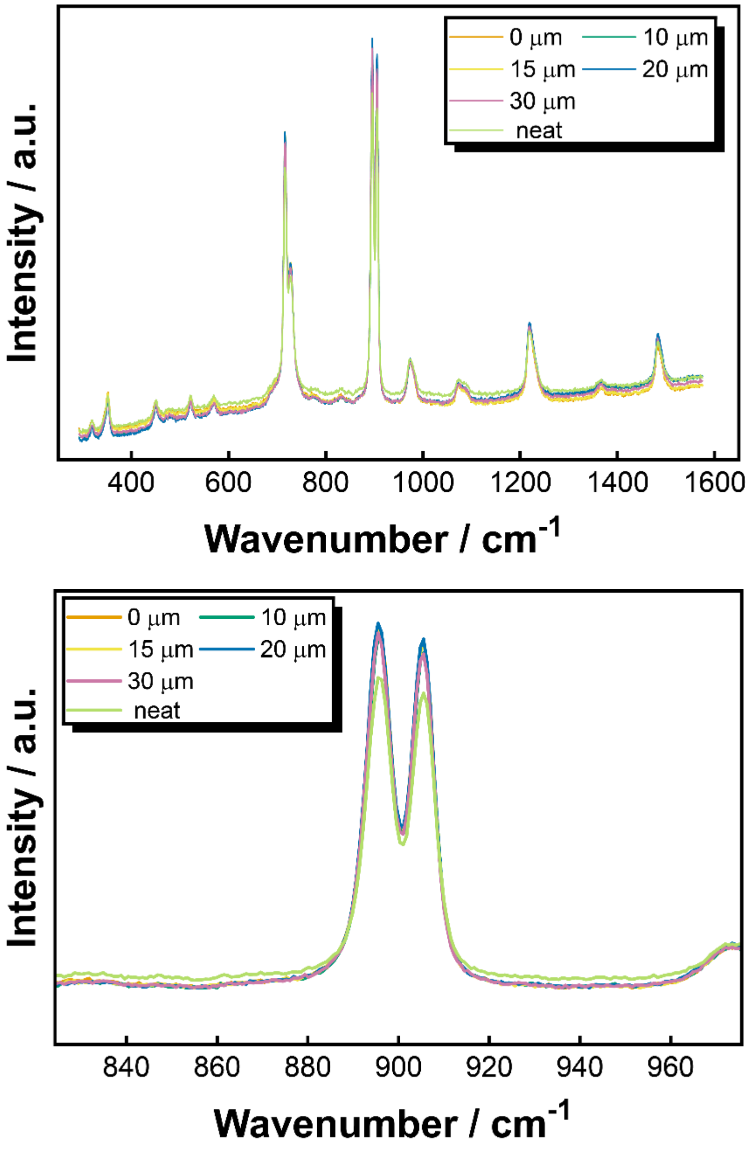

Fig. S6 Raman spectrum for each measurement distance from top of microelectrode.

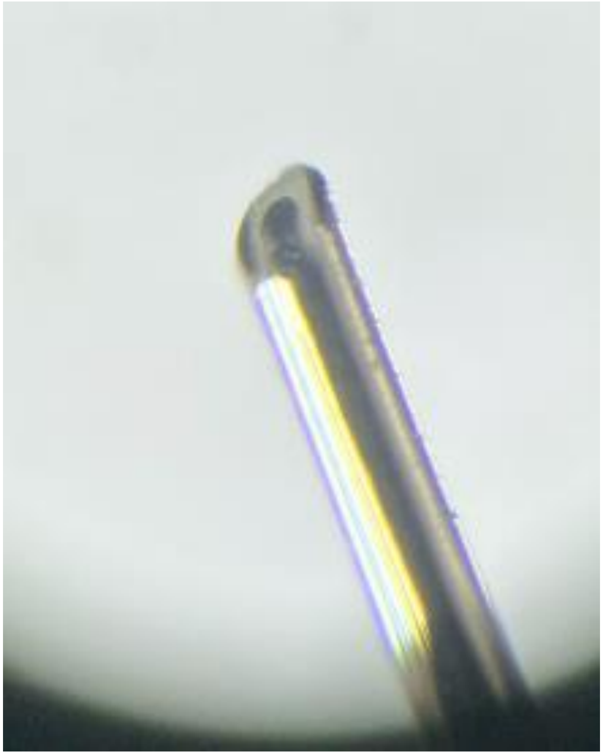

Fig. S7. Optical micrograph of the fabricated microelectrode.
